# Fourier Transform Infrared Spectroscopy in Oral Cancer Diagnosis

**DOI:** 10.3390/ijms22031206

**Published:** 2021-01-26

**Authors:** Rong Wang, Yong Wang

**Affiliations:** School of Dentistry, University of Missouri–Kansas City, Kansas City, MO 64108, USA; wangrong@umkc.edu

**Keywords:** oral cancer diagnosis, oral squamous cell carcinoma, oral dysplasia, Fourier transform infrared spectroscopy, FTIR, infrared imaging, spectral biomarkers, spectral cytopathology, multivariate analysis, machine learning

## Abstract

Oral cancer is one of the most common cancers worldwide. Despite easy access to the oral cavity and significant advances in treatment, the morbidity and mortality rates for oral cancer patients are still very high, mainly due to late-stage diagnosis when treatment is less successful. Oral cancer has also been found to be the most expensive cancer to treat in the United States. Early diagnosis of oral cancer can significantly improve patient survival rate and reduce medical costs. There is an urgent unmet need for an accurate and sensitive molecular-based diagnostic tool for early oral cancer detection. Fourier transform infrared spectroscopy has gained increasing attention in cancer research due to its ability to elucidate qualitative and quantitative information of biochemical content and molecular-level structural changes in complex biological systems. The diagnosis of a disease is based on biochemical changes underlying the disease pathology rather than morphological changes of the tissue. It is a versatile method that can work with tissues, cells, or body fluids. In this review article, we aim to summarize the studies of infrared spectroscopy in oral cancer research and detection. It provides early evidence to support the potential application of infrared spectroscopy as a diagnostic tool for oral potentially malignant and malignant lesions. The challenges and opportunities in clinical translation are also discussed.

## 1. Introduction

Oral cancer is the eighth most common cancer worldwide with an estimated 657,000 new cases and 330,000 deaths annually in 2020, and these numbers are expected to double by 2035 according to the World Health Organization (WHO) [1]. Despite easy access to the oral cavity for examination and significant advances in treatment, oral cancer patients often face very high morbidity and mortality rates due to late-stage diagnosis, which accounts for approximately 70% of all new cases [2]. The 5-year survival rate for oral cancer patients ranges from 20 to 90% depending on the stage of diagnosis [3]. Early-stage oral cancer often manifests as subtle mucosal lesions classified as oral potentially malignant disorders (OPMD) [4,5]. Early detection and effective management of these lesions are critical for improving survival rates and preventing oral cancer progression.

The gold standard for oral cancer diagnosis is biopsy and subsequent histopathological evaluation under a microscope. This process is invasive, time-consuming, and subject to inter-observer variability [6]. Furthermore, histopathological assessment based on tissue morphological alterations does not provide an accurate risk assessment for OPMDs and tends to detect oral cancer at late stages [7]. Various adjunctive techniques have been proposed to facilitate the screening and diagnosis of oral cancer such as exfoliative cytology (cytobrush) [8], vital tissue staining [9], and the use of chemoluminescence or autofluorescence [10,11,12]. However, despite the continuous effort of improvement, most techniques still exhibit limited ability to provide accurate information and help clinicians detect oral cancer in early stage [13]. Recently, molecular markers and salivary tests have been investigated for their potential in early oral cancer detection [14,15,16]. However, so far, no single biomarker can reliably validate the presence or predict the prognosis of oral cancer [17,18].

The search for a fast, simple, accurate, and cost-effective diagnostic method for early oral cancer detection is still underway. One promising technique is Fourier transform infrared (FTIR) spectroscopy, which provides molecular fingerprints of biological samples based on vibrational transitions of chemical bonds in the samples upon interaction with infrared light. FTIR is a non-invasive and label free method that can detect early bimolecular changes associated with a neoplasm condition even before the emergence of morphological abnormalities, which strongly supports its role in early cancer detection [19,20]. To date, considerable research work has demonstrated the competitive to superior performance of FTIR in comparison to conventional cancer screening and diagnostic techniques, making it a potentially powerful clinical tool in modern medicine [21,22,23,24].

## 2. Oral Malignant and Potentially Malignant Disorders

Oral cancer is a malignant condition on the lips or in the oral cavity including the tongue, gingiva, mouth floor, parotid, salivary glands, and throat. More than 90% of oral cancer is oral squamous cell carcinoma (OSCC) [25,26]. Oral carcinogenesis is a highly complex multifactorial process that arises when epithelial cells are affected by different genetic changes. There are several well-known risk factors for oral cancer such as smoking and alcohol consumption. Oral cancer is 2–3 times more prevalent in men than women [27].

The vast majority of oral cancer patients have pre-existing oral lesions called oral potentially malignant disorders (OPMDs) that precede the development of OSCC. OPMDs consist of a group of mucosal lesions associated with higher risk of malignant transformation. The worldwide prevalence rate of OPMDs is estimated to be 4.47% [28]. The most common OPMDs encountered in clinical practice include leukoplakia (white patch), erythroplakia (red patch), lichen planus, and oral submucous fibrosis. While the clinical manifestations of OPMDs are common, it is very hard to predict the outcome for individual cases following the detection of an OPMD [29,30]. The malignant transformation rate for OPMDs was recently reported to be 0.13–34%, with the majority of cases remaining unchanged, becoming enlarged or reduced in size or even resolving completely [31]. Factors associated with an increased malignant transformation risk include gender, lesion site, lesion type, habits (such as alcohol consumption and smoking), and the histologic diagnosis of epithelial dysplasia [5]. The human papillomavirus as a risk factor has also been discussed, but its role remains controversial [32,33,34].

## 3. Current Diagnostic/Grading/Staging/Methods and Limitations

The clinical presentation of OPMDs is subject to further histological evaluation, which results in the diagnosis of hyperplasia, hyperkeratosis, oral epithelial dysplasia (OED), or OSCC [35]. OED is a range of cytological and architectural changes in oral epithelium caused by an accumulation of genetic alterations that is associated with an increased risk of progression to OSCC [36]. An OED can be graded as mild, moderate, or severe, based on the WHO’s three-tier classification system. Grading of OED is used to assess the probability of malignant transformation, with a higher grade indicating a larger chance of malignant transformation [37,38]. There are no clear guidelines with regard to treatment or follow-up for OED. Generally speaking, mild dysplasia is often conservatively managed through watchful waiting, while severe dysplasia may require excision of the lesion and active surveillance for recurrence [39].

Correct diagnosis and timely treatment of OPMDs play an essential role in early oral cancer detection and prevention. However, the WHO’s gold standard grading system for OED has many limitations. First of all, the efficacy and usefulness of histopathological grading of precursor lesions for predicting malignant transformation have long been debated in the literature as malignant transformation can also occur in the absence of OED and histopathologic grading alone is unable to provide a risk assessment for OED [7,40,41,42,43,44]. Secondly, this method is highly subjective, with wide intra- and inter-observer variability in the grading outcomes and poor reproducibility [45,46]. Thirdly, the histopathological evaluation process is time-consuming since every tissue biopsy needs to be manually examined, resulting in delays in patient treatment and care [47].

If OSCC is diagnosed, effective cancer management requires accurate cancer grading to establish suitable treatment plans, estimate the risk of recurrence, and predict patient prognosis. The current grading of OSCC utilizes both the TNM (Tumour, Node, Metastasis) system and histopathologic grading. The internationally accepted TNM system of cancer staging assesses the extent of tumor growth in the whole body based on the size of the primary tumor (T), the involvement of regional lymph nodes (N) as well as distant metastases (M). Meanwhile, the histopathologic grade (G1-G4) of OSCC is established according to tumor histology and cytomorphology of tumor lesions [48]. This multifactorial diagnostic system considers characteristics of the tumor (e.g., differentiation), the tumor–host interface (invasion), and host reactions (inflammation). Unfortunately, the histopathological grading of oral cancer is a subjective process and provides little or no value for predicting prognosis [49].

Therefore, there is an urgent need for a modern diagnostic tool that provides rapid, objective, and accurate diagnosis of OPMDs for early oral cancer detection and prevention, as well as accurate OSCC grading for better oral cancer management

## 4. Fourier Transform Infrared Spectroscopy/Microspectroscopy

### 4.1. Fourier Transform Infrared (FTIR) Fundamentals

FTIR spectroscopy is an established analytical technique with diverse applications. It was traditionally used by chemists to characterize the molecular structures of a material. Molecules have discrete energy levels for electronic transitions, molecular vibrations, and molecular rotations. When a molecule is irradiated by infrared light, it absorbs a certain amount of the incident radiation at a specific energy/frequency and undergoes vibrational excitation from the ground state to a higher vibrational energy state. The unique pattern of infrared absorption by a particular molecule or functional group produces characteristic bands in their FTIR spectra. The band position is affected by the mass of vibration, the type of molecular bond (e.g., single or double bond), the intra- and inter-molecular environment, and the coupling with other vibrations; the band height is proportional to the concentration of corresponding chemical moieties; and the band width provides an estimate of intermolecular interactions. FTIR spectroscopy provides a biochemical profile of proteins, nucleic acids, lipids, and carbohydrates in a biological sample, called “biomolecular fingerprinting” [50,51] and is sensitive enough to probe subtle changes in the molecular structure and microenvironment such as the secondary structure of proteins, the mutation of nucleic acids, and the peroxidation of phospholipids [52,53,54,55,56].

There are three regions for the infrared spectrum: near-infrared (NIR) in the 0.76–2.5 µm (12,500–4000 cm^−1^) region, mid-infrared (MIR) in the 2.5–25 µm (4000–400 cm^−1^) region, and far-infrared in the 25–1000 µm (400–10 cm^−1^). The most commonly used region for biological applications is MIR, which consists of the fingerprint region of 1800–900 cm^−1^ for proteins (amide I/II/III), lipids, carbohydrates, and nucleic acids. NIR spectroscopy may be used in similar applications to MIR spectroscopy. NIR spectra are occupied by overtone (resonant bands above the fundamental bands) and combinational bands with the typical absorption coefficients two orders of magnitude lower than that of MIR fundamental bands. Therefore, NIR light can penetrate much deeper into the sample surface than MIR light, which makes NIR spectroscopy better suited for deep tissue sampling and the examination of highly moist specimens. The disadvantages of NIR spectroscopy include a significantly lower chemical specificity and difficulty in spectral interpretation [57]. Far infrared is considered a promising treatment modality for certain medical conditions such as knee osteoarthritis [58].

### 4.2. FTIR Sample Techniques

There are two major sample techniques for FTIR spectroscopy: transmission and reflection. The transmission technique is a simple technique in which an infrared beam directly passes through a sample, the transmitted energy is measured, and a spectrum is generated. Careful sample preparation is required as the sample needs to be thin enough (a few µm) to avoid absorption signal saturation; the sample has to be dried before FTIR measurements due to strong infrared absorption of water; and special infrared transparent sample holders such as BaF_2_ disks are also required for this technique. In the reflection technique, the infrared beam is reflected from the interface between the sample and another medium such as air and special substrates before detection. The reflection technique is widely used to acquire infrared spectra in a non-destructive way with no sample preparation (e.g., dissolution or thin-film). The reflection technique further consists of internal and external reflections. The most used internal reflection is attenuated total reflection (ATR). In ATR, the infrared beam passes through a crystal with a high refractive index called internal reflection element (IRE) beneath the sample. The wave extends beyond the surface of the crystal and penetrates a small distance (0.5–2 µm) into the sample in the form of an evanescent wave before it returns to the crystal. The evanescent wave will be attenuated due to infrared absorption of the sample and generate a FTIR spectrum. One of the key advantages of ATR is that it requires minimal or no sample preparation before spectral measurements due to the small penetration depth of infrared light into the sample surface. This technique is particularly suitable for measuring samples with high water content. External reflection techniques include specular reflection (smooth sample surface) and diffuse reflection (rough sample surface). If the thickness and/or absorptivity of a sample is not high enough to yield a spectrum with an adequate signal-to-noise ratio, the transflectance technique may be used. In transflectance mode, the sample such as cells is fixed on an infrared reflective element (e.g., low-e slide) and the infrared beam transmitted through the sample is reflected to pass through the sample again before reaching the detector. This technique effectively doubles the optical path for the sample and enhances signal absorbance.

### 4.3. FTIR Microspectroscopy

With a microscope coupled to a FTIR spectrometer, a FTIR microspectroscopy (e.g., imaging) system provides spatially resolved information based on multiple infrared spectra in an array format. In a FTIR image, each individual pixel comprises a full spectrum for the particular sample location and therefore both spectral and spatial information of the sample is integrated into a three-dimensional data hypercube [59]. A conventional FTIR microspectroscopy system consists of a microscope with a focal plane array detector, coupled to a Michelson interferometer-based spectrometer with a broadband globar thermal source. A FTIR microspectroscopy system can be operated in either transmission mode or reflectance/transflectance mode. A transmission FTIR microspectroscopy system requires careful sample preparation: the sample needs to be thin (<10 µm) and flat with a smooth surface to minimize unwanted optical effects such as scattering. On the other hand, a reflectance FTIR microspectroscopy system such as ATR-FTIR has many advantages over its transmission counterpart. In addition to minimal sample preparation, it can also achieve significantly improved spatial resolution (3–4 µm) compared to transmission mode, which allows the detection of the heterogeneous distribution of biomolecules in a biological sample [60]. Detailed information about FTIR spectroscopy and microspectroscopy can be found in a recently published review article by Bec et al. [59].

FTIR spectroscopy and microspectroscopy techniques have the potential to detect early changes in the biochemical content and conformational structure during the carcinogenesis progression. Since the middle of the 20th century, FTIR spectroscopy and microspectroscopy have been studied as label-free, non-invasive, highly sensitive, and specific analytical tools for the detection and characterization of malignancies in a wide variety of tissues including skin, brain, breast, colon, cervix, lung, stomach, ovary, prostate, leukemia, lymphoma, and squamous epithelium [21,61].

### 4.4. Common FTIR Bands for Biomolecules

**Figure 1:**
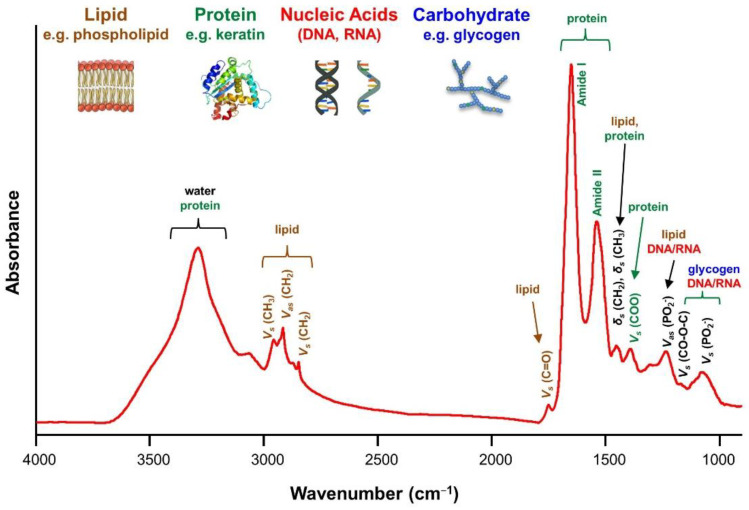
Common Fourier transform infrared (FTIR) bands for biomolecules in oral epithelium.

The FTIR spectrum for a biological sample is a combination of the characteristic absorption bands of all proteins, lipids, nucleic acids, and carbohydrates in the sample [62,63]. The bands of proteins can be assigned to the amino acid side groups or the peptide backbone in the range 1700–1500 cm^−1^. The vibrational modes of the peptide backbone generate amide I and II bands. The amide I band (1700–1600 cm^−1^) is mainly associated with the C=O stretching vibration and the amide II band (1600–1500 cm^−1^) is mainly associated with the bending vibration of the N–H bond. Amides I and II bands are commonly used to investigate the secondary structure of proteins [50]. The bands at 1450 and 1400 are attributable to asymmetric and symmetric methyl bending modes [64]. The spectra of lipids consist of absorption bands in several spectral regions: the region of 3050–2800 cm^−1^ for asymmetric and symmetric stretching vibrations of –CH_2_ and –CH_3_, the region of 1500–1350 cm^−1^ for deformation vibrations of –CH_2_ and –CH_3_ from the lipid acyl chains, the region of 1745–1725 cm^−1^ for symmetric stretching vibration of the ester carbonyl bond (C=O), and the region of 1270–1000 cm^−1^ for asymmetric (1240 cm^−1^) and symmetric (1080 cm^−1^) vibrations of –PO_2_^−^ in phospholipid [65]. The spectra of nucleic acids are characterized in four spectral regions: the region of 1780–1550 cm^−1^ for in-plane vibrations of double bonds of the bases, the region of 1550–1270 cm^−1^ for the deformation vibrations of the bases coupled with the sugar vibrations, the region of 1270–1000 cm^−1^ for vibrations of –PO_2_^−^, and the region of 1000–780 cm^−1^ for the vibrations of the sugar-phosphate backbone [66]. The carbohydrate spectra include bands in the following regions: the region of 3600–3050 cm^−1^ is assigned to the stretching vibration of O–H, the region of 3050–2800 cm^−1^ is assigned to the stretching vibrations of –CH_3_ and –CH_2_, the region of 1200–800 cm^−1^ is assigned to the stretching vibrations of the C–O/C–C groups, and the region of 1500–1200 cm^−1^ is dominated by deformational modes of the CH_3_/CH_2_ groups [67]. Figure 1 illustrates the common FTIR bands for important biomolecules in oral epithelium.

### 4.5. Comparison of FTIR with Other Spectroscopic Diagnostic Techniques

Two other commonly used spectroscopic diagnostic techniques are Raman spectroscopy and fluorescence spectroscopy. Similar to FTIR spectroscopy, Raman spectroscopy also probes the vibrational states of molecules and also has high chemical specificity. However, it is based on the Raman Effect, an inelastic scattering of incident photons by the vibrating molecules. Raman and FTIR spectroscopies are considered complementary. The same vibrations may have different FTIR and Raman activities depending on the symmetry of molecules. For example, the asymmetrical water molecule has a strong infrared absorption but a weak Raman scattering. Therefore, the Raman technique is very useful for the examination of highly moist fresh biological samples. Raman spectroscopy and microspectroscopy are also potent tools in biomedical applications including oral cancer diagnosis [68,69,70,71]. Limitations of the technique include the presence of an intense fluorescence background noise in biological samples, weak signal, poor signal-to-noise ratio, and long acquisition time [72]. As complementary techniques, FTIR and Raman spectroscopies can be used together to provide additional chemical and structural insights.

Unlike FTIR and Raman, fluorescence spectroscopy is based on the spontaneous emission of radiation by a fluorescent molecule (fluorophore) when interacting with the exciting light. Naturally occurring fluorophores include collagen, tryptophan, elastin, keratin, and hemoglobin, etc. Changes in the fluorescence spectra of oral mucosa can be detected and used to help the screening of oral cancer/pre-cancer [73,74]. However, tissue generally contains limited natural fluorophores and their spectroscopic bands are broad and overlapping, which makes it very hard to distinguish them and reduces the specificity of fluorescence spectroscopy for diagnostic applications [6]. Autofluorescence techniques have been scrutinized as a diagnostic adjunct for OPMD and OSCC due to poor study designs and inconclusive results [75,76]. The American Dental Association has recommended against the use of autofluorescence imaging for the assessment of clinically evident oral mucosal lesions [13].

## 5. Signal Preprocessing and Data Analysis

### 5.1. Signal Preprocessing

FTIR spectral/hyperspectral imaging data generally need to be preprocessed first to remove or reduce biochemically irrelevant signal contributions from physical, macro-structural, and environmental factors, in order to improve the accuracy of quantitative data analysis toward disease detection applications. Typical spectral preprocessing includes background subtraction, spectrum region selection, spectral smoothing, light scattering correction, baseline adjustment, normalization, spectral differentiation, and outlier removal [77].

### 5.2. Exploratory Analysis

After signal preprocessing, exploratory analysis is usually employed next to identify biochemical patterns and trends of the data and help understand the nature of the samples, outliers, and experimental errors. There are several ways to do exploratory analysis including univariate analysis, bivariate analysis, and multivariate analysis. Univariate analysis evaluates only a single property (variable) such as the intensity at a given wavenumber, while bivariate analysis evaluates two properties (variables) at the same time. They are commonly used to generate chemical maps of interested functional groups based on band intensities or band ratios [78]. Although univariate and bivariate analyses are easy to use and the resulting chemical maps are also easy to interpret, these techniques make use of only a very small fraction of the available spectral information.

FTIR spectral data often contain thousands of variables (wavenumbers) and measurements (objects/observations), which hold an enormous amount of biochemical information. Particularly, hyperspectral image data are high-dimensional data with a full spectrum at each pixel. In order to extract significant and meaningful information from them, it is often necessary to apply an appropriate multivariate analysis for data interpretation [79]. Multivariate analysis allows the evaluation of several properties (variables) of the spectra or the entire spectra at the same time. The application of multivariate statistical methods to chemistry or biology is also called Chemometrics [80]. Principal component analysis (PCA) is the most widely used multivariate exploratory analysis method. It aims to reduce the complexity of spectral datasets by linearly transforming the original coordinate system into a new coordinate system defined by the principal components that best explain the variance in the dataset. PCA is an unsupervised method frequently used for spectral data dimensionality reduction, which helps extract useful signals from unwanted noise and reduce computational complexity.

### 5.3. Classification Modeling Process

Following exploratory data analysis, a predictive model using experimental FTIR data needs to be built for disease detection. For this purpose, the initial dataset is split into two or three subsets: the training dataset, the validation dataset (optional), and the testing dataset. During the training phase, a classification model is built using the pre-labelled training dataset (e.g., FTIR spectra of normal and pathological cases), so that the different classes are well separated. The model parameters learned during the training phase are stored for further validation. During the testing phase, the unlabeled data (e.g., FTIR spectra from new samples with unknown disease attributes) are classified or predicted using the model built in the training phase. For small-sized datasets, cross-validation is employed using samples from the training set to optimize the model parameters. The most commonly used cross-validation methods include leave-one-out cross-validation (for sample size ≤20), leave-p-out cross-validation, k-fold cross-validation, and continuous-block cross-validation (for replicate spectra). For a large number of samples (>100), a separate validation subset is used to optimize the model.

### 5.4. Clustering and Classification Methods

Unsupervised clustering and/or supervised classification analyses are further employed to separate (cluster) and group (classify) biological samples or FTIR image pixels based on certain similarity measures of the corresponding spectra. These similarity measures are calculated using specific mathematical distance functions such as Euclidean distance, Manhattan distance, and Minkowski distance. Commonly used clustering and discriminant methods for FTIR spectroscopic data analysis include k-means, fuzzy c-means (FCM), hierarchical cluster analysis (HCA), k-nearest neighbors (KNN), support vector machines (SVM), soft independent modeling of class analogy (SIMCA), linear discriminant analysis (LDA), partial least squares discriminant analysis (PLS-DA), artificial neural networks (ANN), and convolutional neural networks (CNN) [77]. Unsupervised clustering analysis helps identify hidden structures in unlabeled datasets and is often used as a precursor to supervised methods, whereas supervised methods build classification models for predicting the disease attributes of new samples based on their spectral profiles.

K-means is the simplest unsupervised clustering method for splitting a spectral dataset into a set of pre-determined k groups. K-means is a “hard” clustering in which each spectrum belongs to only one cluster, while in fuzzy c-means, each spectrum can belong to multiple clusters with different membership grades. Hierarchical clustering is an unsupervised method that generates a set of clusters in a tree-shaped dendrogram, which shows the hierarchical relationship between the clusters. KNN is a local non-parametric supervised method, which classifies a sample spectrum based on the most frequent disease attribute label of the closest k neighboring spectra in a projected feature space. SVM aims to identify a hyperplane in an N-dimensional space (N is the number of variables) that optimally classifies the spectra in the training dataset so that the maximum margin from the boundaries of different classes is achieved. Then, the hyperplane is used as a decision boundary to help classify new spectra; data points falling on either side of the hyperplane can be assigned to different classes. SIMCA is based on PCA modeling performed for each class in the training set. Unknown samples are compared to the PCA class models and assigned to the class according to their analogy with the training samples. LDA is one of the most popular classification methods used for high-dimensional spectral data. LDA is similar to PCA in the sense that both of them look for latent dimensions to explain variance in the data. Unlike PCA, which is an unsupervised method aiming to find new dimensions with the most variations, LDA is a supervised method aiming to maximize the between-class variance and minimize the within-class variance through a linear discriminant function. Other nonlinear variants of LDA include quadratic discriminant analysis, multiple discriminant analysis, and canonical discriminant analysis, in which the same goal is achieved using various non-linear discriminant algorithms. The unsupervised method is often used in conjunction with supervised methods to analyze a very large spectral dataset. One of the most powerful combinations is PCA-LDA, in which the training spectral dataset is first reconstructed using PCA to reduce the number of variables (dimension reduction), and then the reconstructed data are fed into the LDA classifier for performing classification.

Regression analysis is the statistical processes for estimating the relationship between the observable variables (measured FTIR spectral data) and the predicted variables (categorical disease attributes). Two commonly used regression methods are partial least square (PLS) regression and principal components regression (PCR). PLS aims to find a linear regression model using a latent variable approach by projecting the predicted variables and the observable variables to a new space, while PCR aims to find hyperplanes of maximum variance between the two variables. When the predicted variables are categorical (e.g., 0 and 1 for normal case and cancer), PLS is called partial least square discriminant analysis (PLS-DA). OPLS-DA is a modification of the classical PLS-DA, which usually performs better than PLS-DA. Similar to PCA-LDA, a PLS-LDA is a combinational method that first uses a PLS model to reduce the original spectral variables to a small number of latent variables, and then applies the LDA to classify the samples [81].

When data complexity increases, ‘black box’ algorithms (i.e., unknown classification rules) can be applied such as artificial neural networks (ANN), random forests, and deep-learning approaches. ANNs are computational models inspired by the functionality of the central nervous system of the human brain, in which many nodes called artificial neurons are arranged in layers and each node is connected to all other nodes in adjacent layers. A typical ANN is made up of one input layer, one output layer, and several hidden layers. The more hidden layers, the deeper the neural network. A random forest utilizes multiple decision trees to provide a more accurate and stable prediction. Deep learning approaches offer a great variety of opportunities for solving classical imaging tasks and also for state-of-the-art problem-solving in the spatial–spectral domain. A CNN is a class of deep neural networks with the ability to learn spatial characteristics of a FTIR image. Its ability to process both spectral and spatial information significantly improves the classification performance for FTIR hyperspectral image data [82]. These methods all have nonlinear classification nature and can provide higher classification accuracy for highly complicated spectral data. However, they also require more modeling parameters to be optimized and higher computational power [81].

### 5.5. Model Performance Validation

To discriminate the diseased from the healthy cases is the ultimate goal of every medical diagnostic procedure. An ideal diagnostic test has the ability to completely discriminate subjects with and without diseases. Unfortunately, such a perfect test does not exist in real life. A realistic test comprises true positive (TP), false positive (FP), true negative (TN), and false negative (FN) cases. Diagnostic accuracy of a test is defined by its ability to discriminate between the diseased and healthy cases and can be calculated by the proportion of true positive and true negative in all evaluated cases $\left(\frac{\mathrm{TP}+\mathrm{TN}}{\mathrm{TP}+\mathrm{FP}+\mathrm{TN}+\mathrm{FN}}\right)$.

Diagnostic accuracy can also be quantified by other merit measures such as sensitivity and specificity. Sensitivity is defined as the probability of getting a positive test result in subjects with the disease $\left(\frac{\mathrm{TP}}{\mathrm{TP}+\mathrm{FN}}\right)$. Specificity is defined as the probability of getting a negative test result in subjects without the disease $\left(\frac{\mathrm{TN}}{\mathrm{TN}+\mathrm{FP}}\right)$ [83]. The performance of any classification model must be evaluated with a validation dataset or a testing dataset using quality merits such as accuracy, sensitivity, and specificity.

## 6. FTIR for Oral Cancer Diagnosis

FTIR spectroscopy has been shown by many studies to be a prospective novel diagnostic approach for various types of cancers due to its ability to distinguish cancer samples from normal ones at high sensitivity, specificity, and accuracy [24]. In this section, twenty-two studies on the applications of various FTIR techniques in oral cancer/precancer research are reviewed. All results summarized in this section are human studies in the MIR region unless otherwise noted.

### 6.1. Oral Tissue Studies

The fact that thin tissue samples as prepared for histological examination are readily suitable for infrared spectroscopic analysis conveniently promotes the integration of FTIR techniques into existing histopathological diagnostic routines. About two decades ago, several groups applied FTIR spectroscopy and mapping to study biochemical differences between normal and malignant oral tissues. Schultz et al. revealed that poorly-differentiated OSCC cells produce a relatively homogeneous and distinctly abnormal cell biochemistry, whereas well-differentiated epithelial cells present a highly heterogeneous distribution of cellular components and suggested that FTIR analysis of cell components (e.g., DNA and keratin) could be used to distinguish cancerous tissues from normal epithelial structures [84,85]. Fukuyama et al. observed a series of FTIR spectral differences between normal and malignant oral tissues including bands related to keratin, collagen, phosphate of nucleic acids, and membrane phospholipids. Specifically, FTIR spectral differences between OSCC and normal gingival epithelium were observed in the band regions of 1482–1431 cm^−1^ and 1274–1183 cm^−1^. The shoulder at 1368 cm^−1^ disappeared in OSCC, and the bands at 1246 and 1083 cm^−1^ found in the normal gingival epithelium shifted to 1242 and 1086 cm^−1^ in OSCC, respectively [86]. Wu et al. used fiber-optic ATR spectroscopy on freshly-cut human oral tissues and found that the 1745 cm^−1^ band for the ester group (C=O) vibration of triglycerides, the C–H stretching bands between 3100 and 2800 cm^−1^, and the amide I band at 1646 cm^−1^ were good markers for distinguishing normal oral tissue from malignant ones [87].

From 2003 to 2013, one research group from Italy published a series of papers on their studies of normal, pre-cancerous, and cancerous tissues of the oral cavity by collecting reflectance FTIR spectra of thin tissue sections on a steel support. Bruni et al. observed distinct FTIR chemical maps of vibrational bands at 970 cm^−1^ (DNA), 1026 cm^−1^ (collagen), 1550 cm^−1^ (proteins), and 1735 cm^−1^ (lipids) between normal and diseased oral tissues and reported that proliferating and regressive states of the tumors could be identified via the presence of a high content of DNA or collagen, respectively [88]. Conti et al. continued the investigation using supervised and unsupervised multivariate analyses (HCA and PCA) and showed that changes in vibrational frequency and intensity of proteins and nucleic acids as well as the visualization of single wavenumber or band ratio images enabled a qualitative and quantitative evaluation of the changes in the proliferating activity from dysplastic to neoplastic states [89,90]. Sabbatini et al. further conducted vibrational analysis of both epithelial and connective tissue sections of OSCC at various malignancy grades (G1–G3) and identified a series of potential spectral markers for OSCC grading including the increase in unsaturated lipid chains, evidencing the occurrence of acyl chain peroxidation process changes in the protein structure with increased helical conformations and longer side chains, a significant carbohydrate consumption due to enhanced cellular activity, an elevated level of free glycogen associated with carcinogenesis, structural alterations in nucleic acids with a higher degree of DNA methylation, and an increased amount of RNA indicating more cellular transcriptional activity. They also suggested that spectroscopic biochemical changes occurred in both cancerous epithelium and neighboring connective structures [91]. The investigations by this group showed satisfactory agreement between the vibrational results and the histopathological results and contribute to biochemical understandings of these lesions toward an early diagnosis.

Meanwhile, Pallua et al. investigated microarrays of OSCC tissues using FTIR imaging and found that the use of multivariate methods HCA and KMC (k-means clustering) in spectral regions of 3650–3050 cm^−1^, 3000–2800 cm^−1^, and 1750–850 cm^−1^ considerably increased the information content of the infrared datasets. Their results indicate that intra-operative and surgical specimens of the oral cavity can be characterized by FTIR microscopic imaging [92]. Banerjee et al. applied FTIR spectroscopy in the differentiation of oral leukoplakia and OSCC histological tissues using linear and quadratic support vector machine (SVM) at 10-fold cross-validation. Six spectral features (1782, 1713, 1665, 1545, 1409, and 1161 cm^−1^) were obtained through the Feature Forward Selection method, achieving a classification between leukoplakia and OSCC with 81.3% sensitivity, 95.7% specificity, and 89.7% overall accuracy. The biochemical assignments of these spectral features revealed changes in glycogen and keratin content between leukoplakia and OSCC histological sections. This study not only supports the development of FTIR spectral markers in cancer research, but also reveals its clinical promise for disease classification and risk assessments of oral lesions [93]. Naurecka et al. investigated the differences of FTIR-ATR and FT-Raman spectroscopy among leukoplakia, oral cancer, and normal tissues. FTIR spectral differences were observed at 1238 cm^−1^ (related to phosphate stretching in nucleic acids) between normal and cancer tissues and at 1030 cm^−1^ (related to –CH_2_OH vibration in glycogen) among normal, leucoplakia, and cancer tissues [94].

### 6.2. Oral Cell Studies (FTIR Cytopathology)

FTIR cytopathology is a novel approach for cancer/pre-cancer screening by studying the biochemical composition of exfoliated cells using FTIR. FTIR cytopathology provides rapid measurement of cellular biochemistry and identifies reproducible spectral patterns that exist in disease states. One research group from the United States conducted a series of investigations on the application of FTIR cytopathology to cancer screening using exfoliated cells from the oral cavity. Papamarkakis et al. demonstrated that FTIR spectra of squamous cells from the tongues of healthy people could be differentiated from those collected from patients with oral diseases (e.g., dysplastic and cancer cases) by using the unsupervised PCA method. The spectra of normal, dysplastic, and cancerous cells demonstrated a gradual change that allowed reliable detection of oral cancer. These spectral changes can be attributed to the variations in the chemical composition of their corresponding cells [20]. Miljković et al. from the same group further trained an ANN to automatically distinguish the clinical oral disease cases from the normal cases, achieving sensitivity and specificity values of 96% and 94.3%, respectively [95]. Similar results (sensitivity of 95.5% and specificity of 94.7%) were achieved for exfoliated esophageal cells [96]. The authors concluded that infrared cytopathology of exfoliated cells from the oral cavity is a method of superior sensitivity for detecting oral diseases. Notably, the findings from this group indicate that FTIR cytopathology can detect biochemical changes in morphologically normal cells from patients with pre-cancerous oral diseases, providing strong support for the use of FTIR in early oral cancer detection [97].

Recently, Ghosh et al. investigated the integration of FTIR and Raman spectroscopy in the discrimination of exfoliated oral cells from oral cancer and pre-cancer (leukoplakia) patients as well as healthy volunteers with and without smoking habit. The PCA-LDA model of the dual spectra yielded a classification accuracy of 98% compared to the accuracy of 85% and 82% from FTIR or Raman alone, respectively, in a spectrum-wise comparison. When the mean of all spectra from a patient was used, the overall classification efficiency was 73%, 80%, and 87% for FTIR, Raman, and combined approaches, respectively. Their study demonstrates the capability of FTIR and Raman spectroscopy of oral exfoliated cells individually and jointly, together with chemometric analysis for the screening and prediction of oral cancer among the susceptible population [98].

FTIR spectroscopy has also been used for drug–cell interaction studies. Giorgini et al. conducted in vitro FTIR microspectroscopy analysis of primary OSCC cells under controlled hydrated conditions treated with two chemotherapy drugs cisplatin and 5-fluorouracil. The data evidenced meaningful spectroscopic differences due to alterations in cellular proteins, lipids, and nucleic acids and revealed the different drug pathways and extents of cellular damage that were not provided by traditional cell-based assays [99].

Chiu et al. demonstrated a wax-physisorption-based kinetic analysis method for the detection of oral precancer and cancer using synchrotron-based infrared microspectroscopy. Linear discriminant analysis (LDA) of oral cell FTIR spectra produced an accuracy rate of up to 89.6% for discriminating normal cells from cancer cells using methylene (CH_2_) and methyl group (CH_3_) stretching vibrations in the range of 3000–2800 cm^−1^. A lower absorbance ratio of ν_as_ CH_2_/ν_as_ CH_3_ was found in more advanced cancerous samples than that of normal ones. Their findings suggest that cancerous oral samples can be differentiated from normal ones based on different wax physisorption properties caused by the polarity and structure of molecules on the cell surface [100].

### 6.3. Biofluid Studies

Biofluids (e.g., blood including plasma and serum, saliva, sputum, urine, and tears) can provide a systemic snapshot of the human body. As a diagnostic medium, they offer considerable advantages due to their non-/minimally invasive collection at a low cost. Vibrational spectroscopic analysis of biofluids offers effective diagnosis via specific spectral markers and has been reported in the detection of a variety of cancers including ovarian [101], colorectal [102], lung [103], brain [104], and breast cancer [105].

Menzies et al. demonstrated that ATR-FTIR can be used to discriminate sputum samples of oral cancer patients from the control. A feature selection method based on partial least squares (SlimPLS) was used to determine significant wavenumbers (1650 cm^−1^, 1550 cm^−1^, and 1042 cm^−1^) for cancer and normal sputum discrimination, and those spectral features suggested changes to protein and glycoprotein structures within sputa cells [106].

Oral submucous fibrosis (OSF) is found to have the highest malignant potential among all pre-cancerous oral lesions. Rai et al. used FTIR spectroscopy together with chemometric techniques to differentiate the serum metabolic signatures of OSF patients from healthy controls. Multivariate statistical techniques (PCA, HCA, PLS-DA) achieved excellent separation of OSF spectra from normal ones using nineteen significant wavenumbers (*p* ≤ 0.001) between 1725 cm^−1^ and 1020 cm^−1^, representing alterations in lipids, proteins, carbohydrates, and nucleic acids. These findings suggest that FTIR spectroscopy combined with chemometric analysis can be potentially employed for rapid and accurate preoperative screening and diagnosis of OSF [107].

Adeeba et al. profiled plasma samples from oral cancer patients and “niswar” (a dipping tobacco product) users using ATR-FTIR. Chemometric analysis of the data revealed a clear separation among the groups. PLS-DA and OPLS-DA models provided a classification rate of 87.7% and 89.5%, respectively. Their results indicate that FTIR spectroscopy coupled with chemometric analysis can be used for the preliminary discrimination of plasma samples of oral cancer patients, “niswar” users, and healthy individuals [108].

Recently, extracellular vesicles (EVs) have attracted considerable interest in cancer research. EVs are small membrane-bound vesicles naturally released from cells including tumor cells and immune cells into plasma, urine, saliva, and other biofluids. EVs have been shown to function in almost every step of cancer progression. Cancer EVs contain a unique biomolecular cargo consisting of proteins, nucleic acids, and lipids. Through the analysis of this specific cargo, biomarkers have been identified and developed for cancer diagnosis and prognosis [109]. EVs offer an exciting opportunity to improve our understanding of oral cancer biology that may translate to improved clinical practice [110].

Zlotogorski-Hurvits et al. assessed the diagnostic potential of FTIR spectra of salivary exosomes (a type of EV) and showed that a specific IR spectral signature for oral cancer salivary exosomes could be accurately differentiated from that for healthy individuals, based on small changes in the conformations of proteins, lipids, and nucleic acids with optimized artificial neural networks. Specifically, IR spectra of oral cancer were consistently different from healthy ones at 2924 cm^−1^ and 2854 cm^−1^ (membrane lipids), 1543 cm^−1^ (transmembrane proteins), and 1072 cm^−1^ (nucleic acids). The PCA-LDA model successfully classified the samples with a sensitivity of 100%, a specificity of 89%, and an accuracy of 95%. The SVM model showed a training accuracy of 100% and a cross-validation accuracy of 89% [111].

### 6.4. Tumor Microenvironment Study

The application of FTIR in oral cancer diagnosis has generally focused on spectral changes in the epithelium. However, recent studies have demonstrated that the biochemical changes in the extracellular matrix may also have the potential to be biomarkers for cancer [112,113,114]. Ukkonen et al. employed FTIR imaging to study the biological changes of the tumor microenvironment caused by the human tongue SCC and melanoma cells using a 3D organotypic myoma model. Their results suggest that the features present in the amide and collagen triplet region (1700–1600 cm^−1^) could serve as spectral markers for cancer-induced modifications in the tumor microenvironment [115].

A summary of all the reviewed studies in this section can be found in Table 1.

## 7. Clinical Translation of FTIR

**Figure 2:**
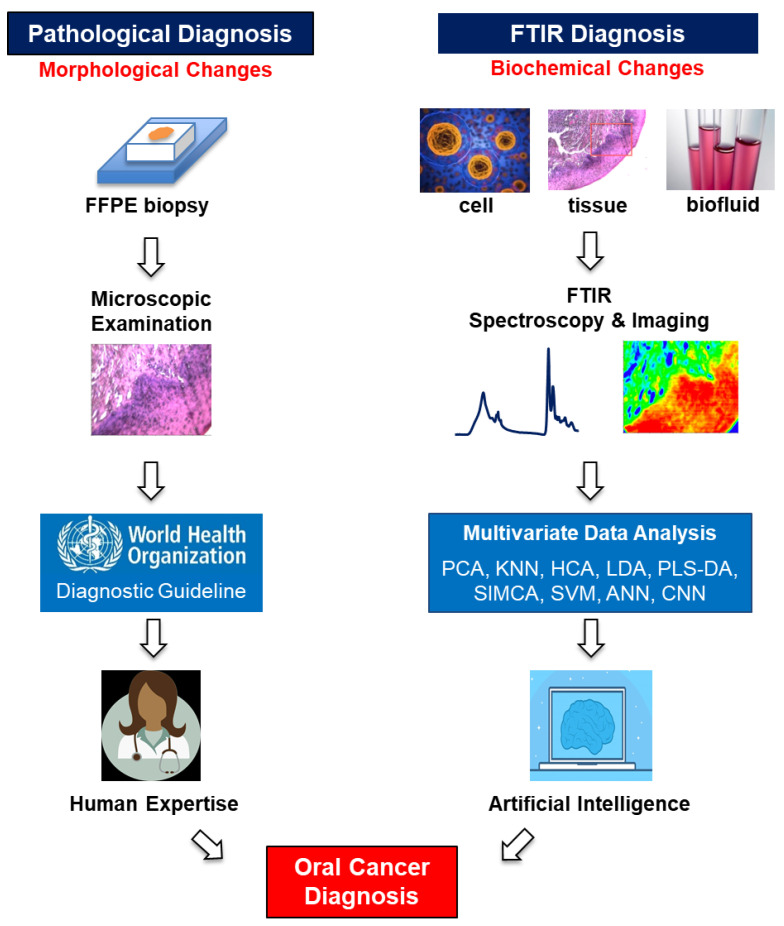
Comparison between pathological and FTIR oral cancer diagnoses.

FTIR is a versatile analytical tool that works with tissues, cells, or body fluids. The FTIR imaging technique in transmission mode can be readily implemented to complement conventional histopathological biopsy diagnosis. The FTIR cytopathology technique in reflection/transflection mode proves to be a valuable tool in exfoliated cell analysis. Additionally, the ATR-FTIR technique is very useful in non-invasive biofluid analysis. Compared to the century-old pathological diagnostic method, FTIR diagnosis is based on biochemical changes underlying the disease pathology rather than morphological changes of tissue, and offers high sensitivity, specificity, and accuracy in cancer detection. FTIR combined with big data technologies such as multivariate statistical analyses and machine learning offer tremendous potential for the prevention, early detection, and management of oral cancer. A comparison between the traditional pathological diagnosis and the novel FTIR diagnosis for oral cancer is illustrated in Figure 2.

Various sample techniques face their own challenges such as beam scattering artifacts (Mie scattering) for the transmission mode of tissue sampling, the coffee-ring effect for ATR mode of biofluid sampling, and the electric-field standing wave effect for reflection/transflection mode of cell or tissue sampling [116,117,118]. Most of the challenges have been addressed by optimal sample preparation and the use of advanced spectral preprocessing techniques like extended multiplicative signal correction [119,120].

Clinical implementation has been impeded by practical hurdles like the low speed of data acquisition, low sample throughput, unsatisfactory spatial resolution, issue of clinical integration process, and lack of optimized computational procedures for rapid extraction of clinically useful information [121]. There have been some latest technological developments to reduce these barriers.

The demand for high throughput and speed has led to the development of discrete frequency imaging systems using high brightness quantum cascade laser (QCL) technology, capable of producing high-resolution images in a fraction of the time of the traditional FTIR system [122,123]. For example, an advanced confocal FTIR instrument was recently developed by S. Mittal et al. using refractive infrared optics and a QCL source. This instrument provides simultaneously high resolution (2-μm pixel size) and high signal-to-noise ratio (>1300) as well as a speed increase of ~50-fold for obtaining classified results compared with the present FTIR imaging technique. It was demonstrated that clinical biopsies of a typical patient can be analyzed in 1 h and about 100 tissues can be analyzed in a day with this newly developed FTIR instrument [124]. Another type of high-throughput ATR-FTIR technology has been developed using patented silicon internal reflection elements (Si IREs) along with machine learning technology for the analysis of serum and demonstrated a sensitivity of 93.2% and a specificity of 92.8% for differentiating brain cancer patients and control [125].

The spatial resolution of a traditional bench-top FTIR imaging system with a conventional globar source is about 20 µm × 20 µm. The use of a highly brilliant synchrotron light source can improve the spatial resolution to a few µm [79]. However, a synchrotron source has limited availability and accessibility. Recent developments in high-resolution infrared microscope optics have led to even better spectral quality and spatial resolution with a conventional source [126]. Superior spatial resolution can also be achieved with near-field IR techniques, which have the potential to break the diffraction limit constraints for a 100-fold improvement in spatial resolution [127].

Another practical barrier to be addressed in order to employ FTIR in clinical practice is concerning the sample substrate. FTIR microspectroscopy in transmission mode is performed using thin tissue sections on a special substrate highly transparent in the mid-infrared region such as barium fluoride (BaF_2_) or calcium fluoride (CaF_2_) substrates, which are expensive to use and fragile to handle. The transflection sampling modality utilizes a transparent glass slide with an infrared reflective layer. Although they are cheap and robust, concerns have been expressed for distortions arising from the electric-field standing wave effect. Efforts have been made to address the substrate issues. Bassan et al. proposed the use of standard glass slides for FTIR imaging in transmission mode. Although they are mostly opaque in the mid-infrared region, there is a narrow spectral region of 3800–2500 cm^−1^ with sufficient transmission to enable accurate analysis of N–H, O–H, and C–H stretching bands. Excellent classification accuracies (98.25% for epithelium, 99.94% for stroma, 100.00% for blood, and 97.22% for necrosis) have been achievable between malignant and non-malignant epithelium using standard glass slides [128]. To address the mentioned issues and to better incorporate FTIR to the existing histopathological workflow, one study applied FTIR to coverslip-covered stained histological tissues received from the pathologist on standard glass slides and demonstrated classification accuracies over 95% among normal epithelium, malignant epithelium, normal stroma, and cancer associated stroma using a random forest classification model [129].

Other advancements in technologies are further accelerating the adoption of FTIR in oral cancer and other biomedical areas. For example, three-dimensional FTIR imaging has been developed to meet the needs of modern pathology. Ogunleke et al. demonstrated a high-throughput infrared microscopy method that utilized automated image correction followed by spectral analysis for 3D FTIR image reconstruction. The new approach enabled quantitative metabolic parameter extraction from the FTIR spectra for the characterization of the brain tumor metabolism [130]. Metasurface-enhanced infrared reflection spectroscopic cytopathology has been developed that utilizes plasmonic metasurfaces to localize and intensify the evanescent field near the cell’s membrane, and to conduct spectroscopic interrogations of the cells attached to the metasurface using reflected infrared light. This approach has a super high sensitivity that a very small part (~50 nm deep) of the cell can generate valuable diagnostic information. Early findings indicate that this approach can effectively differentiate cancerous human colon cells from normal ones [131]. Large-scale clinical translation of FTIR could be expedited by the availability of economic miniaturized instrumentation. Single-chip in-silicon spectrometers enabled by fundamental technological breakthroughs have already been reported [132]. On-chip FTIR spectroscopy employing complementary metal-oxide semiconductor compatible thin-film waveguides and microfluidics has shown good promise as highly integrated, compact, and robust tools for biofluid analysis [133].

Before widespread clinical adoption happens, protocol standardization and extensive clinical trials need to be carried out. Standardization of sample collection, handling, and storage is critical for achieving experimental reproducibility within and among laboratories. Large patient cohorts with different grades of various oral cavity cancers will help validate the technique.

In a recent review, Rai et al. suggested that modern “Omics” strategies (genomics, transcriptomics, proteomics, and metabolomics) can make a significant influence on the identification of molecular biomarkers for early oral cancer detection [134]. As an additional “Omics”, FTIR spectromics should be integrated with other patient information to achieve early oral cancer detection and provide a potential pathway to precision medicine and personalized care in oral cancer treatment and management.

## 8. Conclusions

The studies reviewed in the current paper strongly support the great promise of FTIR as a novel diagnostic tool for early oral cancer detection and management. FTIR spectroscopy has been investigated in the analysis of a variety of oral cancer related biological samples including oral tissues, oral cells, and biofluids. It has also been used to study the oral tumor microenvironment as well as the effects of anti-cancer drugs. The biochemically based FTIR diagnostic approach offers several advantages over the morphologically based gold standard histopathology, among which the most notable benefit is its ability to detect pre-cancerous changes at early stages. The wealth of biochemical and structural information contained in FTIR spectra and images can be fully extracted using various multivariate analysis and machine learning techniques. FTIR in conjunction with chemometric data analysis has demonstrated high sensitivity, specificity, and accuracy in differentiating pathological oral cases from normal ones. Recent technological breakthroughs and advancements in infrared sources, waveguides, detectors, chip integrations, and software development further expedite the clinical translation of FTIR as a fast, economic, accurate, and automated diagnostic system. Integrated with other modern biomedical technologies, FTIR is expected to play a significant role in the early detection of oral cancers in the near future.

## Figures and Tables

**Table 1:** A summary of the oral cancer studies using Fourier transform infrared (FTIR) spectroscopy discussed in this review.

Type of Study	Title of Study	References
	Biochemical imaging and 2D classification of keratin pearl structures in oral squamous cell carcinoma	[84]
	In situ infrared histopathology of keratinization in human oral/oropharyngeal squamous cell carcinoma	[85]
	A study on the differences between oral squamous cell carcinomas and normal oral mucosas measured by Fourier transform infrared spectroscopy	[86]
	Distinguishing malignant from normal oral tissues using FTIR fiber-optic techniques	[87]
	Histological and microscopy FT-IR imaging study on the proliferative activity and angiogenesis in head and neck tumors	[88]
Oral tissue studies	FT-IR microscopy imaging on oral cavity tumors, II.	[89]
	Microimaging FT-IR of oral cavity tumors. Part III: Cells, inoculated tissues and human tissues.	[90]
	Infrared microspectroscopy of Oral Squamous Cell Carcinoma: Spectral signatures of cancer grading	[91]
	Fourier transform infrared imaging analysis in discrimination studies of squamous cell carcinoma	[92]
	Fourier-transform-infrared-spectroscopy based spectral-biomarker selection towards optimum diagnostic differentiation of oral leukoplakia and cancer	[93]
	FTIR-ATR and FT-Raman Spectroscopy for Biochemical Changes in Oral Tissue	[94]
	Spectral cytopathology: new aspects of data collection, manipulation and confounding effects	[95]
	Infrared micro-spectroscopy for cyto-pathological classification of esophageal cells	[96]
Oral cell studies	Cancer Screening via Infrared Spectral Cytopathology (SCP): Results for the Upper Respiratory and Digestive Tracts	[97]
	Chemometric analysis of integrated FTIR and Raman spectra obtained by non-invasive exfoliative cytology for the screening of oral cancer	[98]
	In vitro FTIR microspectroscopy analysis of primary oral squamous carcinoma cells treated with cisplatin and 5-fluorouracil: a new spectroscopic approach for studying the drug-cell interaction	[99]
	Oral cancer diagnostics based on infrared spectral markers and wax physisorption kinetics	[100]
	Fourier transform infrared for noninvasive optical diagnosis of oral, oropharyngeal, and laryngeal cancer	[106]
	Serum-based diagnostic prediction of oral submucous fibrosis using FTIR spectrometry	[107]
Biofluid studies	A comparative profiling of oral cancer patients and high risk niswar users using FT-IR and chemometric analysis	[108]
	Extracellular vesicles in head and neck cancer: A potential new trend in diagnosis, prognosis, and treatment	[110]
	FTIR-based spectrum of salivary exosomes coupled with computational-aided discriminating analysis in the diagnosis of oral cancer	[111]
Tumor microenvironment study	Changes in the microenvironment of invading melanoma and carcinoma cells identified by FTIR imaging	[112]

## Data Availability

No new data were created or analyzed in this study. Data sharing is not applicable to this article.
